# Clinical Outcomes and Immune-Related Adverse Events in Metastatic Non-Squamous NSCLC Treated with First-Line Pembrolizumab–Chemotherapy: A Real-World Study from Serbia

**DOI:** 10.3390/curroncol33050267

**Published:** 2026-05-06

**Authors:** Zlatan Bojić, Filip Marković, Milica Kontić

**Affiliations:** 1 Clinic for Pulmonology, University Clinical Centre of Serbia, 11000 Belgrade, Serbia; zlatan.bojic@kcs.ac.rs (Z.B.); milica.kontic@kcs.ac.rs (M.K.); 2 Faculty of Medicine, University of Belgrade, 11000 Belgrade, Serbia

**Keywords:** immune-related adverse events, immune checkpoint inhibitors, non-small-cell lung cancer, pembrolizumab, real-world evidence

## Abstract

**Background**: Pembrolizumab combined with pemetrexed–platinum chemotherapy is the standard first-line treatment for patients with metastatic non-squamous non-small-cell lung cancer (NSCLC) and a PD-L1 tumor proportion score (TPS) of 1–49%. Real-world data on treatment outcomes and the prognostic relevance of immune-related adverse events (irAEs) in this population remain limited, particularly in Eastern Europe. **Methods**: We conducted a retrospective, single-center real-world study including patients with metastatic non-squamous NSCLC and PD-L1 TPS of 1–49% treated with first-line pembrolizumab plus pemetrexed–platinum chemotherapy between June 2024 and May 2025. Progression-free survival (PFS) was estimated using the Kaplan–Meier method. Cox proportional hazards models were used to evaluate factors associated with PFS, including baseline clinical characteristics and organ-specific irAEs graded according to CTCAE v5.0. **Results**: A total of 107 patients were included. Median PFS for the entire cohort was 7.03 months (95% CI, 4.81–9.26). Immune-related adverse events occurred in 52 patients (48.6%), with thyroid (21.5%) and skin (13.1%) irAEs being the most frequent. The majority of irAEs were grade 1–2, while grade 3–4 events were rare (4.7%) and limited to hepatic toxicity and pneumonitis, all leading to treatment discontinuation. In multivariate analysis, ECOG performance status 2 was independently associated with inferior PFS (HR 3.09, 95% CI 1.74–5.48; *p* < 0.001). Conversely, the occurrence of thyroid irAEs (HR 0.36, 95% CI 0.17–0.78; *p* = 0.009) and skin irAEs (HR 0.28, 95% CI 0.10–0.79; *p* = 0.016) was independently associated with prolonged PFS, whereas pulmonary, hepatic, and gastrointestinal irAEs were not. **Conclusions**: In this real-world cohort of patients with metastatic non-squamous NSCLC treated with first-line pembrolizumab–chemotherapy, clinical outcomes were consistent with prior real-world experience. These findings suggest that low-grade, manageable immune toxicities may serve as pragmatic on-treatment markers of benefit. However, given the retrospective design, limited sample size, and the absence of time-dependent analyses, these associations should be interpreted with caution and considered hypothesis-generating rather than causal.

## Simple Summary

Pembrolizumab combined with platinum-based chemotherapy is the standard first-line treatment for patients with metastatic non-squamous non-small-cell lung cancer (NSCLC) and intermediate PD-L1 expression (1–49%). However, treatment outcomes in routine clinical practice remain heterogeneous, and predictors of benefit are not well defined. In this real-world study from Serbia, we evaluated clinical outcomes and immune-related adverse events (irAEs) in patients treated with first-line pembrolizumab–chemotherapy. We found that patients who developed skin or thyroid irAEs experienced longer progression-free survival. These findings suggest that certain low-grade, manageable immune toxicities may reflect effective immune activation and could serve as practical on-treatment markers of benefit in patients receiving chemo-immunotherapy.

## 1. Introduction

Lung cancer remains the leading cause of cancer-related mortality worldwide, accounting for nearly one in five cancer deaths annually [1]. Non-small-cell lung cancer (NSCLC) comprises approximately 80–85% of all lung cancer cases, and most patients are diagnosed with advanced or metastatic disease, for which long-term survival remains limited despite therapeutic advances [2].

The introduction of immune checkpoint inhibitors (ICIs) targeting the programmed death-1/programmed death-ligand 1 (PD-1/PD-L1) pathway has substantially improved outcomes for patients with advanced NSCLC [3]. Pembrolizumab, a monoclonal antibody against PD-1, has demonstrated significant survival benefits across multiple treatment settings and PD-L1 expression subgroups, both as monotherapy and in combination with platinum-based chemotherapy [4,5,6,7]. As a result, pembrolizumab-based regimens are now widely incorporated into first-line treatment algorithms for metastatic NSCLC without actionable driver alteration [3].

For patients with non-squamous NSCLC and intermediate PD-L1 expression (tumor proportion score [TPS] 1–49%), pembrolizumab combined with pemetrexed and platinum chemotherapy represents the standard first-line treatment approach, based on consistent survival benefits observed in randomized clinical trials [7]. Nevertheless, outcomes in this population remain heterogeneous, and a substantial proportion of patients experience limited or short-lived benefit, highlighting the need for improved characterization of treatment outcomes in routine practice.

Randomized clinical trials enroll highly selected patient populations and may not fully reflect the clinical complexity encountered in everyday oncology practice [8]. Real-world studies therefore play a crucial role in complementing trial data by providing insight into treatment effectiveness, safety, and patterns of care in broader, unselected populations. This is particularly relevant for immunotherapy-based combination regimens, where patient fitness, comorbidities, disease burden, and treatment accessibility may substantially influence outcomes [9].

Despite the growing body of real-world data globally, evidence from Eastern Europe remains limited. Serbia, which has one of the highest lung cancer incidence rates worldwide, is notably underrepresented in published real-world studies evaluating immunotherapy outcomes [10]. Regional differences in healthcare infrastructure, patient demographics, disease characteristics, and access to innovative therapies may influence treatment delivery and clinical outcomes, underscoring the need for region-specific investigations.

Immune checkpoint inhibitors are associated with a distinct spectrum of immune-mediated toxicities, referred to as immune-related adverse events (irAEs), which most commonly involve the skin, endocrine organs, gastrointestinal tract, liver, and lungs [11]. While irAEs are an important consideration in clinical management, their prognostic relevance in patients receiving pembrolizumab–chemotherapy combination for non-squamous NSCLC remains incompletely defined, particularly in real-world settings.

In this context, we conducted a real-world study evaluating clinical outcomes in patients with metastatic non-squamous NSCLC and PD-L1 TPS 1–49% treated with first-line pembrolizumab in combination with pemetrexed–platinum chemotherapy at a tertiary academic center in Serbia. The primary objective was to characterize progression-free survival in routine clinical practice, with secondary objectives including assessment of treatment response, safety, and exploratory evaluation of clinical factors associated with outcomes.

## 2. Materials and Methods

The study included patients with histologically confirmed non-squamous metastatic NSCLC with PD-L1 TPS 1–49% that started treatment between June 2024 and May 2025 with combination therapy that consisted of pembrolizumab and pemetrexed–platinum for the first four cycles and then pembrolizumab and pemetrexed maintenance. The data cutoff date was 1 September 2025. The median follow-up duration was 9.3 months (range 0.1–12.3). Patients lost to follow-up were censored at the date of last known clinical contact. Missing data were minimal for variables included in the analysis and were handled using complete-case analysis. Vital status was determined from institutional electronic health records, including hospital documentation and recorded follow-up visits. In addition, patient status was cross-checked through the national health insurance system, where termination of health insurance coverage may indicate death. The study was conducted at a single academic institution in Serbia. Patients were identified through a prospectively maintained institutional lung cancer registry, which represents a structured clinical database derived from electronic health records (EHRs) of patients treated at our center. The registry is routinely updated and cross-validated against the EHR to ensure completeness and accuracy. Data collection included demographic characteristics, smoking status, ECOG performance status, tumor characteristics, molecular testing results, treatment details, occurrence and type of immune-related adverse events, and survival outcomes. Data were extracted from the EHR, including both structured data fields and unstructured clinical documentation (e.g., radiology reports), by trained investigators and cross-checked for accuracy. All data were de-identified prior to analysis.

All patients underwent routine PD-L1 testing performed on formalin-fixed, paraffin-embedded histology or cytology samples using PD-L1 monoclonal antibodies (22C3 clone by DAKO, Glostrup, Denmark) prior to initiating the first line of treatment. Patients were tested for EGFR mutations by Cobas^®^ EGFR Mutation Test v2 and ALK rearrangements by immunohistochemistry prior to first-line treatment initiation.

Patients with detected driver oncogenes were excluded, so none of the included patients had a known driver oncogene. Response assessments were based on routine radiological evaluations interpreted according to RECIST v1.1 principles. Imaging was typically performed every 8–12 weeks or as clinically indicated [12]. Toxicity was graded according to the Common Terminology Criteria for Adverse Events (CTCAE) v5.0 [13].

### 2.1. Ethics Approval

Data on patients with lung cancer were retrospectively extracted from institutional electronic health records, including demographic, pathological, molecular, treatment, and survival data of patients diagnosed and treated at the University Clinical Centre of Serbia. All data were collected and analyzed in an anonymized manner. The study was conducted in accordance with the Declaration of Helsinki and approved by the Ethics Committee of the University Clinical Centre of Serbia (2268/3; 4 December 2025).

### 2.2. Statistical Analysis

Descriptive methods were used on the demographic characteristics of patients. Baseline information is presented as the number of patients and percentages. Median progression-free survival (PFS) was calculated as the time from the start of therapy until disease progression. Patients still alive on the last day of follow-up were censored. Median PFS was estimated by the Kaplan–Meier method and compared by the log-rank test. Univariable and multivariable Cox proportional hazards regression models were used to calculate hazard ratios (HRs) and confidence intervals (CIs). In the univariate analysis, covariates included age, sex, smoking status (current or former vs. never-smoker), ECOG PS (0–1 vs. 2), the presence of brain metastasis at baseline (yes vs. no), radiotherapy during treatment (yes vs. no), occurrence of thyroid immune-related adverse events (yes vs. no), occurrence of cutaneous immune-related adverse events (yes vs. no), occurrence of gastrointestinal immune-related adverse events (yes vs. no), occurrence of hepatic immune-related adverse events (yes vs. no), and occurrence of pneumonitis as an immune-related adverse event. Multivariate analysis included variables with a significance level of *p* < 0.10 in the univariate analysis. The chi-square test was used to determine the association between response to treatment and the occurrence of immune-related adverse events. Calculated *p*-values were two-sided. We used SPSS v26 for statistical analysis.

## 3. Results

### 3.1. Patient Characteristics

Between June 2024 and May 2025, 107 patients with metastatic non-squamous NSCLC and PD-L1 TPS 1–49% treated with first-line pembrolizumab in combination with pemetrexed–platinum chemotherapy were included. Baseline demographic and clinical characteristics are summarized in Table 1. The mean age at treatment initiation was 68.8 years (range, 48–90). Most patients were male (60.7%) and ever-smokers (86.0%). ECOG performance status (PS) was 0–1 in 72.9% of patients, while 27.1% had ECOG PS 2. Baseline CNS metastases were present in 20.6% of patients, and 23.5% received radiotherapy during systemic treatment. The mean number of metastatic sites was 1.9, with the majority of patients presenting with fewer than three metastatic sites (93.3%).

### 3.2. Treatment Response and Progression-Free Survival

**Figure 1:**
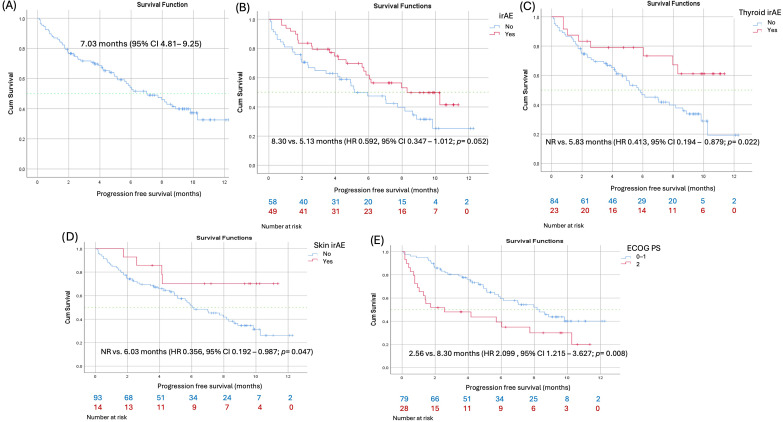
Kaplan–Meier analysis of progression-free survival (PFS) for the entire study cohort (**A**); patients with versus without immune-related adverse events (irAEs) (**B**); patients with versus without thyroid irAEs (**C**); patients with versus without skin irAEs (**D**); and patients with ECOG performance status 0–1 versus 2 (**E**).

Treatment response according to RECIST v1.1 is shown in Table 1. Progressive disease was observed in 29.0% of patients, stable disease in 46.7%, partial response in 21.5%, and complete response in 2.8%. The real-world disease control rate was 71.0%, while the real-world objective response rate was 24.3%. Median progression-free survival (PFS) for the entire cohort was 7.03 months (95% CI, 4.81–9.26) (Figure 1).

### 3.3. Immune-Related Adverse Events

Overall, 52 patients (48.6%) experienced at least one immune-related adverse event (irAE), while 11 patients (10.3%) developed more than one irAE (Table 1). Thyroid irAEs were the most frequently reported (n = 23; 21.5%), followed by skin-related irAEs (n = 14; 13.1%), gastrointestinal irAEs (n = 12; 11.2%), hepatic irAEs (n = 10; 9.3%), and pneumonitis (n = 10; 9.3%).

According to CTCAE v5.0, immune-related adverse events were predominantly grade 1–2, occurring in 47 of 52 patients with irAEs (90.4%), whereas grade 3–4 irAEs were uncommon (5/52 patients, 9.6%; 5/107 overall, 4.7%), consisting exclusively of hepatic toxicity (3/52, 5.8%; 2.8% overall) and pneumonitis (2/52, 3.8%; 1.9% overall); in all cases, grade 3–4 irAEs resulted in permanent treatment discontinuation as per the ESMO Guidelines for the management of toxicities from immunotherapy [14].

### 3.4. Baseline Characteristics According to irAE Status

Comparisons of baseline clinical characteristics between patients who developed irAEs and those who did not are presented in Table 2. No statistically significant differences were observed with respect to sex, smoking status, ECOG PS, baseline CNS metastases, or radiotherapy exposure during treatment (all *p* > 0.05).

Baseline clinical characteristics according to the occurrence of thyroid irAEs and skin irAEs are shown in Table 3 and Table 4, respectively. No significant associations were identified between baseline variables—including sex, smoking status, ECOG PS, CNS metastases, or radiotherapy—and the development of thyroid or skin irAEs (all *p* > 0.05).

### 3.5. Factors Associated with Progression-Free Survival

Results of univariate and multivariate Cox proportional hazards regression analyses for PFS are summarized in Table 5. In univariate analysis, ECOG PS 2 was associated with significantly shorter PFS compared with ECOG PS 0–1 (HR 2.10, 95% CI 1.22–3.63; *p* = 0.008). The occurrence of thyroid irAEs (HR 0.41, 95% CI 0.19–0.88; *p* = 0.022) and skin irAEs (HR 0.36, 95% CI 0.19–0.99; *p* = 0.047) was associated with improved PFS. Other variables were not significantly associated with PFS.

In multivariate analysis (Table 5), ECOG PS 2 remained independently associated with inferior PFS (HR 3.09, 95% CI 1.74–5.48; *p* < 0.001). Both thyroid irAEs (HR 0.36, 95% CI 0.17–0.78; *p* = 0.009) and skin irAEs (HR 0.28, 95% CI 0.10–0.79; *p* = 0.016) retained an independent association with prolonged PFS.

## 4. Discussion

In our study, patients with metastatic non-squamous NSCLC and PD-L1 TPS 1–49% treated with first-line pembrolizumab in combination with pemetrexed–platinum chemotherapy achieved a median progression-free survival (PFS) of 7.03 months (95% CI: 4.81–9.26). This outcome was lower than that reported in the PD-L1 TPS 1–49% subgroup of the pivotal KEYNOTE-189 trial, in which median PFS was 8.8 months [7]. Similar to observations from other real-world settings, this difference likely reflects the broader inclusion of patients with less favorable baseline characteristics, such as poorer ECOG performance status and higher disease burden, who are typically underrepresented or excluded from randomized clinical trials.

Importantly, multiple real-world studies evaluating pembrolizumab–chemotherapy combinations in patients with PD-L1 TPS < 50% have reported median PFS estimates comparable to those observed in our cohort. In particular, a Portuguese real-world study of patients with metastatic non-squamous NSCLC treated with first-line pembrolizumab plus pemetrexed–platinum chemotherapy reported a median PFS of approximately 6.7 months, closely mirroring our results [15]. Additionally, the Italian PEMBROREAL study reported a median real-world PFS of 8.0 months in a similar patient population, further supporting that outcomes observed in routine clinical practice tend to be modestly attenuated compared with registrational trial benchmarks while remaining consistent across different healthcare systems [9]. Real-world outcomes are shaped not only by patient- and disease-related factors but also by system-level differences across healthcare settings. In Serbia and similar resource-constrained environments, limited access to comprehensive molecular profiling, variability in imaging intervals, and differences in treatment availability and sequencing may influence both patient selection and clinical outcomes. These factors should be considered when interpreting our results in the context of data from Western European or North American cohorts, where healthcare systems are typically more standardized.

In our cohort, ECOG performance status 0–1, skin immune-related adverse events, and thyroid immune-related adverse events were independently associated with prolonged progression-free survival, whereas the occurrence of immune-related adverse events considered as a composite variable was not. This pattern is biologically and methodologically plausible and has been highlighted in prior analyses. As discussed by Socinski et al., aggregating heterogeneous irAEs into a single composite endpoint may obscure clinically meaningful associations, as different irAE organ systems reflect distinct immunological mechanisms, severities, and clinical consequences [16]. In particular, low-grade cutaneous and endocrine irAEs are thought to represent sustained immune activation compatible with continued treatment exposure, whereas visceral irAEs (e.g., pneumonitis, hepatitis) are more likely to prompt treatment interruption, corticosteroid use, or hospitalization, thereby attenuating any potential association with improved outcomes. Consequently, evaluating organ-specific irAEs rather than irAEs as a unified category may provide a more accurate reflection of the relationship between immune activation and therapeutic benefit.

The association of skin-related irAEs with prolonged PFS in our cohort aligns with the broader concept that cutaneous toxicity may represent a clinically visible marker of on-treatment immune activation that remains compatible with continued systemic therapy [16]. In first-line chemo-immunotherapy trials, rash and pruritus are among the most frequently observed immune-mediated or immune-associated toxicities, reflecting the skin as a common “target organ” of PD-1/PD-L1–driven autoimmunity. For example, in the KEYNOTE-189 trial, rash was among the most common adverse events reported with pembrolizumab plus pemetrexed–platinum chemotherapy, underscoring the high incidence of cutaneous events in this setting and mirroring the pattern observed in our cohort [7].

A similar rationale applies to thyroid immune-related adverse events, which represent one of the most common endocrine toxicities associated with immune checkpoint blockade and are readily identifiable through routine laboratory monitoring [17,18]. Thyroid dysfunction often develops early during treatment, is typically manageable with standard hormone replacement therapy, and rarely necessitates permanent treatment discontinuation, making it particularly suitable as an on-treatment marker of immune engagement [19]. In a recent real-world analysis of advanced lung cancer patients treated with immune checkpoint inhibitors, Liu et al. reported that endocrine irAEs—predominantly thyroid dysfunction—were associated with higher response rates and favorable survival outcomes, supporting their prognostic relevance in routine clinical practice [20]. Likewise, in a real-world cohort of patients receiving chemo-immunotherapy for metastatic NSCLC, Trudu et al. identified thyroid dysfunction as one of the most frequent irAEs and observed numerically prolonged PFS among patients who developed endocrine toxicity [21]. Together with our findings, these data suggest that thyroid irAEs, similar to skin irAEs, may reflect a sustained yet clinically manageable immune activation state associated with improved disease control in patients treated with pembrolizumab-based chemo-immunotherapy.

Real-world chemo-ICI data further support a link between irAEs and improved outcomes, although the strength and consistency of this association vary according to irAE phenotype and severity. In a multi-institutional retrospective series of NSCLC patients treated with immunotherapy plus chemotherapy, Morimoto et al. reported that the occurrence of irAEs was associated with overall clinical benefit; notably, skin reactions tended to be associated with improved outcomes, although this did not reach statistical significance in their smaller cohort. Importantly, the authors emphasized that mild (grade 1–2) irAEs were the category most consistently associated with favorable survival outcomes [22].

In line with these findings, analyses of real-world chemo-immunotherapy cohorts suggest that the prognostic relevance of irAEs is organ-specific rather than uniform. In the Spinnaker first-line chemo-immunotherapy real-world study, thyroid irAEs (single-organ**)** were significantly associated with longer PFS and OS, whereas skin irAEs were associated with improved OS but did not reach statistical significance for PFS. Similar to our cohort, pulmonary and hepatic irAEs were over-represented among grade 3–4 toxicities and did not demonstrate a favorable prognostic pattern.

Consistently, large pooled analyses of atezolizumab-containing chemo-immunotherapy trials in NSCLC have demonstrated that the survival benefit associated with irAEs is organ-specific rather than uniform across all toxicities [16]. In these analyses, cutaneous and endocrine irAEs, including thyroid dysfunction, were associated with improved survival outcomes, whereas pulmonary (pneumonitis) and hepatic irAEs did not confer a similar prognostic advantage—likely reflecting their greater clinical severity, higher likelihood of treatment interruption, and need for systemic immunosuppression. In agreement with these observations, in our cohort, skin irAEs and thyroid dysfunction were associated with favorable PFS, while pulmonary and hepatic irAEs were not [16].

In our cohort, patients with poor performance status (ECOG PS 2) experienced significantly worse clinical outcomes, consistent with extensive evidence identifying ECOG performance status as one of the strongest prognostic factors in advanced non-small-cell lung cancer. Across real-world studies, impaired functional status has been repeatedly associated with reduced progression-free and overall survival in patients receiving immune checkpoint inhibitors, either as monotherapy or in combination with chemotherapy [23,24,25,26,27,28]. Several biological and clinical mechanisms may underlie this observation, including diminished immune competence, increased systemic inflammation, a higher comorbidity burden, and a reduced ability to tolerate or sustain prolonged systemic treatment [24,29].

Although our findings support an organ-specific prognostic role of immune-related adverse events and confirm the importance of performance status in routine clinical practice, they should be interpreted with caution. The real-world observational design limits causal inference and may introduce selection- and treatment-related biases. Accordingly, the observed associations are best regarded as hypothesis-generating and should be contextualized within the methodological limitations discussed below.

## 5. Limitations

Several limitations of the present study should be acknowledged. First, the retrospective design and single-center setting may introduce selection bias and limit the generalizability of our findings. Second, the relatively modest sample size limits the statistical power of subgroup analyses, particularly for less frequent immune-related adverse events such as pneumonitis and hepatic toxicity, as well as for analyses according to radiotherapy type, timing, or clinical indication. Third, although we explored associations between immune-related adverse events (irAEs) and clinical outcomes, we did not apply landmark or time-dependent analyses; therefore, the observed associations may be influenced by time-dependent bias, including immortal time bias, and should be interpreted as hypothesis-generating rather than causal.

Furthermore, molecular testing was limited to EGFR and ALK alterations, as broader genomic profiling was not routinely available during the study period, which may have resulted in the incomplete exclusion of rare oncogenic drivers. In addition, several potentially relevant variables—including comorbidity indices (e.g., Charlson Comorbidity Index), detailed comorbidity data (such as chronic pulmonary disease or diabetes mellitus), socioeconomic factors, nutritional status, and circulating biomarkers (e.g., inflammatory cytokines such as IL-6 and TNF-related pathways, as well as metabolic markers such as adiponectin)—were not systematically collected in routine clinical practice and were therefore unavailable for analysis.

Radiotherapy was delivered in heterogeneous clinical contexts without standardized documentation of dose, fractionation, or timing relative to systemic therapy, introducing potential confounding by indication. Similarly, treatment exposure variables, including the total number of administered cycles and treatment duration, were not available in a structured format, limiting our ability to assess their potential impact on outcomes. Vital status was determined from institutional electronic health records and cross-checked using national health insurance data. However, deaths occurring outside the hospital system may not have been fully captured in real time. Overall survival data were also immature at the time of analysis.

Despite these limitations, our study provides important real-world insights into the outcomes of pembrolizumab–chemotherapy in an underrepresented population from Eastern Europe. Serbia has one of the highest lung cancer incidence rates worldwide, yet it remains scarcely represented in immunotherapy real-world datasets. Our findings therefore complement existing evidence by reflecting treatment effectiveness and toxicity patterns in routine clinical practice, including patients with poorer performance status and comorbidities who are frequently excluded from randomized trials.

Notably, our results support the emerging concept that the prognostic relevance of irAEs in NSCLC is organ-specific rather than uniform, particularly in the chemo-immunotherapy setting. The independent association of skin and thyroid irAEs with prolonged progression-free survival, coupled with the absence of benefit for pulmonary, gastrointestinal, and hepatic irAEs, is biologically plausible and may reflect differences in both immune activation patterns and treatment continuity. Low-grade, treatment-compatible toxicities may serve as markers of sustained immune engagement, whereas severe visceral toxicities, often requiring treatment interruption and immunosuppressive therapy, may attenuate potential clinical benefit.

## 6. Conclusions

In this real-world cohort of patients with metastatic non-squamous NSCLC and PD-L1 TPS 1–49% treated with first-line pembrolizumab plus pemetrexed–platinum chemotherapy, outcomes were consistent with previously reported real-world experience, and ECOG performance status remained a key determinant of clinical benefit.

The occurrence of skin and thyroid immune-related adverse events was independently associated with prolonged progression-free survival, whereas pulmonary, gastrointestinal, and hepatic irAEs were not. However, given the retrospective design, potential selection bias, and the absence of time-dependent or landmark analyses, these findings should be interpreted with caution and regarded as hypothesis-generating. Prospective studies with appropriate methodological approaches are needed to confirm the observed associations.

## Figures and Tables

**Table 1:** Baseline demographic and clinical characteristics of the study population.

N = 107	N (%)
Mean age at treatment start (range) [years]	68.8 (48–90)
Sex	
Male	65 (60.7)
Female	42 (39.3)
Smoking status	
Ever-smoker	92 (86.0)
Never-smoker	15 (14.0)
ECOG PS	
0–1	78 (72.9)
2	29 (27.1)
Radiotherapy during treatment	
Yes	30 (23.5)
No	77 (76.5)
Number of metastatic sites	
Mean	1.9
3<	81 (93.3)
3≥	26 (6.7)
CNS metastasis at baseline	
Yes	22 (20.6)
No	85 (79.4)
Patients who experienced immune-related adverse events	52 (48.6)
Patients who experienced >1 immune-related adverse event	11 (10.3)
Thyroid immune-related adverse events	23 (21.5)
Skin immune-related adverse events	14 (13.1)
Gastrointestinal immune-related adverse events	12 (11.2)
Hepatic immune-related adverse events	10 (9.3)
Pneumonitis immune-related adverse events	10 (9.3)
Best response	
^α^PD	31 (29.0)
SD	50 (46.7)
PR	23 (21.5)
CR	3 (2.8)
Real-world DCR	71.0%
Real-world ORR	24.3%
Median PFS (95% Confidence Interval) [months]	7.03 (4.812–9.255)

**Table 2:** Comparison of baseline clinical characteristics between patients who experienced at least one immune-related adverse event (irAE) and those without irAEs during treatment.

	Patients Without irAE (55)	Patients with irAE (52)	*p* Value
**Sex**			0.22
Male	37 (67.3%)	28 (53.8%)	
Female	18 (32.7%)	24 (46.2%)	
**Smoking status**			0.50
Current or former	49 (89.1%)	43(82.7%)	
never-smoker	6 (10.9%)	9 (17.3%)	
**CNS metastasis**			0.29
No	41 (74.5%)	44 (84.6%)	
Yes	14 (25.5%)	8 (15.4%)	
**ECOG PS**			0.29
0–1	43 (78.2%)	44 (84.6%)	
2	12 (21.8%)	8 (15.4%)	
**Radiotherapy**			0.75
Yes	17 (30.9%)	13 (25.0%)	
No	38 (69.1%)	39 (75.0%)	

**Table 3:** Association between baseline clinical characteristics and the occurrence of thyroid immune-related adverse events.

	Patients Without Thyroid irAE (84)	Patients with Thyroid irAE (23)	*p* Value
**Sex**			0.17
Male	55 (65.5%)	10 (43.5%)	
Female	29 (34.5%)	13 (56.5%)	
**Smoking status**			0.24
Current or former	74 (88.1%)	18(78.3%)	
never-smoker	10 (11.9%)	5 (21.7%)	
**CNS metastasis**			0.75
No	66 (78.6%)	19 (82.6%)	
Yes	18 (21.4%)	4 (17.4%)	
**ECOG PS**			0.20
0–1	63 (75.0%)	15 (65.2%)	
2	20 (25.0%)	8 (34.8%)	
**Radiotherapy**			0.88
Yes	25 (29.8%)	5 (21.7%)	
No	59 (70.2%)	18 (78.3%)	

**Table 4:** Association between baseline clinical characteristics and the occurrence of skin immune-related adverse events (irAEs).

	Patients Without Skin irAE (93)	Patients with Skin irAE (14)	*p* Value
**Sex**			0.08
Male	59 (63.4%)	6 (42.9%)	
Female	34 (36.6%)	8 (57.1%)	
**Smoking status**			0.57
Current or former	80 (86%)	12 (85%)	
never-smoker	13 (14%)	2 (15%)	
**CNS metastasis**			0.75
No	74 (79.6%)	11 (78.6%)	
Yes	19 (20.4%)	3 (21.4%)	
**ECOG PS**			0.19
0–1	70 (75.2%)	8 (57.1%)	
2	23 (24.8%)	6 (42.9%)	
**Radiotherapy**			0.86
Yes	26 (28.0%)	4 (28.6%)	
No	67 (72.0%)	10 (71.4%)	

**Table 5:** Univariate and multivariate Cox proportional hazards regression analyses for progression-free survival (PFS).

	Univariate Regression Analysis			Multivariate Regression Analysis		
	HR	95% CI	*p*	HR	95% CI	*p*
Sex (male vs. female)	1.287	0.762–2.172	0.395			
CNS metastasis (Yes vs. No)	1.226	0.646–2.328	0.534			
Radiotherapy (Yes vs. No)	1.117	0.638–1.954	0.150			
Smoking status (ever vs. never-smoker)	1.112	0.778–1.588	0.560			
ECOG PS (2 vs. 0–1)	2.099	1.215–3.627	0.008	3.089	1.740–5.482	<0.001
Thyroid irAE (yes vs. no)	0.413	0.194–0.879	0.022	0.360	0.167–0.775	0.009
Skin irAE (yes vs. no)	0.356	0.192–0.987	0.047	0.279	0.099–0.792	0.016
Gastrointestinal irAE (yes vs. no)	1.283	0.606–2.713	0.515			
Hepatic irAE (yes vs. no)	0.693	0.251–1.917	0.480			
Pneumonitis (irAE) (yes vs. no)	0.390	0.175–1.199	0.116			

## Data Availability

The dataset generated and analyzed during the current study is not publicly available due to privacy regulations and consent restrictions but is available from the corresponding author upon reasonable request. All data shared will be de-identified in accordance with ethical guidelines.
